# DNA Methylation and Gene Expression of Matrix Metalloproteinase 9 Gene in Deficit and Non-deficit Schizophrenia

**DOI:** 10.3389/fgene.2018.00646

**Published:** 2018-12-11

**Authors:** Ju Gao, Hongwei Yi, Xiaowei Tang, Xiaotang Feng, Miao Yu, Weiwei Sha, Xiang Wang, Xiaobin Zhang, Xiangrong Zhang

**Affiliations:** ^1^Department of Geriatric Psychiatry, Nanjing Brain Hospital, Affiliated to Nanjing Medical University, Nanjing, China; ^2^Centers of Disease Prevention and Control for Mental Disorders, Shanghai Changning Mental Health Center, Shanghai, China; ^3^Department of Pharmacology, School of Medicine, Southeast University, Nanjing, China; ^4^Department of Psychiatry, Affiliated WuTaiShan Hospital of Medical College, Yangzhou University, Yangzhou, China; ^5^Department of Psychiatry, Nanjing Qing Long Mountain Psychiatric Hospital, Nanjing, China; ^6^Medical Psychological Institute of the Second Xiangya Hospital, Central South University, Changsha, China

**Keywords:** deficit schizophrenia, matrix metalloproteinase-9, DNA methylation, gene expression, negative symptoms, pyrosequencing

## Abstract

The biological pathology of deficit schizophrenia (DS) remains unclear. Matrix metalloproteinase 9 (MMP9) might be associated with neural plasticity and glutamate regulation, involved in schizophrenia pathogenesis. This study explores gene expression and DNA methylation of MMP9 in peripheral blood mononuclear cells (PBMCs) and their relationship with clinical symptoms in DS and non-deficit schizophrenia (NDS). Pyrosequencing was used to determine DNA methylation at CpG sites in exon 4 and exon 5 of *MMP9* in 51 DS patients, 53 NDS patients and 50 healthy subjects (HC). RT-qPCR was used to detect *MMP9* expression. Clinical symptoms were assessed by BPRS, SANS and SAPS scales. *MMP9* expression in PBMCs was significantly higher in DS than NDS and HC subjects. Compared to NDS patients, DS patients had significantly lower DNA methylation at individual CpG sites in exon 4 and exon 5 of *MMP9*. Correlation analysis showed that DNA methylation in exon 4 was negatively correlated with gene expression in DS group. Positive correlation was found between *MMP9* expression and negative symptoms in total schizophrenic patients. The social amotivation factor of SANS and negative syndrome of BPRS was negatively correlated with DNA methylation of CpG5-1 in DS patients but not in NDS patients. DS patients showed a specific abnormality of peripheral *MMP9* expression and DNA methylation, indicating a pathological mechanism underlying DS as a specific subgroup of schizophrenia.

## Introduction

Schizophrenia is a severe psychiatric disorder with impairment of perception, thought, emotion and behavior, resulting in profoundly impaired social function. Negative symptoms are characterized by an absence or reduction of affective, social and behavioral expression, which are regarded as important predictors of treatment response and prognosis (Foussias et al., 2011). However, the heterogeneity of negative symptoms, primary or secondary symptoms, could lead to the substantial differences in clinical outcome of antipsychotic response and disease prognosis (Aleman et al., 2016). Deficit schizophrenia (DS), proposed by Carpenter et al. (1988), characterizes patients with primary and permanent negative symptoms including restricted affect, diminished emotional range, poverty of speech, curbing of interests, diminished sense of purpose and diminished social drive (Carpenter et al., 1988; Kirkpatrick et al., 1989, 2001). Increasing evidence demonstrates discrepant factors between DS and non-deficit schizophrenia (NDS) in clinical symptoms, disease course, genetic variation (Hong et al., 2005; Wonodi et al., 2006; Bakker et al., 2007; Rethelyi et al., 2010), neuroimaging changes (Lahti et al., 2001; Galderisi et al., 2008; Voineskos et al., 2013; Lei et al., 2015) and neuropsychology (Cohen et al., 2007; Yu et al., 2015; Tang et al., 2016), suggesting that DS might be a homogeneous disease entity with a unique pathogenesis. The research on DS might help to understand the etiology of negative symptoms and prediction biomarkers for the long-term prognosis of patients with schizophrenia.

The matrix metalloproteinase, a large family of extracellular proteolytic enzymes, is implicated in numerous developmental and disease-related processes (Sternlicht and Werb, 2001). Matrix metalloproteinase-9 (MMP9) is the best-characterized MMP family member and is thought to have an important role in the pathophysiology of neuropsychiatric disorders, such as schizophrenia, bipolar disorder, stroke, neurodegeneration and brain tumors (Vafadari et al., 2015). Evidence from animal models demonstrated that extracellular proteolytic activity of MMP9 can remodel the synaptic microenvironment, which might be involved in mechanisms of long-term potentiation (LTP), learning and memory and brain disease involving aberrant plasticity (Lepeta and Kaczmarek, 2015). Michaluk et al. (2011) revealed excessive MMP9 caused elongation and thinning of dendritic spines in the hippocampal neurons in both *in vivo* and *in vitro* models (). Interestingly, it was reported MMP9 regulated surface trafficking of *N*-methyl-D-aspartate receptors (NMDAR) in rat hippocampal neurons *in vitro* (Michaluk et al., 2009); these receptors are importantly related to cellular and molecular processes involved in synaptic adaptation, plasticity and NMDAR-dependent neuronal pathologies (Lau and Zukin, 2007; Groc et al., 2009).

Previous reports on MMP9 in patients with schizophrenia are inconsistent. Rybakowski et al. (2009) genotyped the functional -1562C/T polymorphism of *MMP9* and found a significant preponderance of C/C genotype and C allele in the schizophrenia group compared to normal controls. Domenici et al. (2010) detected increased MMP9 and TIMP-1 (tissue inhibitor of matrix metalloproteinases-I) in peripheral blood of patients with schizophrenia (). Moreover, a randomized double-blind clinical trial reported that minocycline, whose pleiotropic effects include decreasing the expression and activity of MMP9 (Yao et al., 2004), acted as an efficient adjuvant therapy to benefit negative symptoms in early schizophrenia (Chaudhry et al., 2012). However, Niitsu et al. (2014) reported that neither serum mature BDNF nor MMP9 levels differed between chronic schizophrenia and controls, a finding confused by unmatched smoking status between groups. Yamamori et al. (2013) observed a significantly increased MMP9 protein in treatment-resistant schizophrenia treated with clozapine, but no association was found between MMP9 and clinical variables. These reports showed no clear consistency regarding the role of MMP9 in schizophrenia, which might be attributed to the various clinical and methodological factors such as variation of psychiatric symptoms severity and the heterogeneity of schizophrenia. Therefore, it would be of value to investigate whether there is a specific involvement of MMP9 in DS patients with prominent and enduring negative symptoms.

Epigenetics refers to the combination of mechanisms that confer long-term and heritable changes in gene expression without altering the DNA sequence itself. DNA methylation is one of the most common epigenetic alterations which influences genomic expression through methylation of cytosine at C-phosphate-G (CpG) dinucleotides located in distinct genomic regions such as the gene promoter (Deaton and Bird, 2011). In postmortem brains or peripheral blood samples of patients with schizophrenia, DNA methylation alterations in the promoters of candidate genes such as *GELN, COMT, GABRB2* have been reported (Abdolmaleky et al., 2005, 2006; Pun et al., 2011). Genome-wide analysis revealed that monozygotic twins for schizophrenia showed characteristic DNA methylation alterations in peripheral blood samples, highlighting an additional role for epigenetic processes in mediating susceptibility (Dempster et al., 2011). However, it is unclear whether *MMP9* expression might be associated with epigenetic changes that potentially influence negative symptoms in schizophrenia. In the present study we have performed a pyrosequencing approach to detect methylation status of an exonic region of *MMP9* rich in CpG sites in DS, NDS and health control (HC) groups. Furthermore, potential correlations between gene expression, DNA methylation and clinical symptoms were studied. Our primary hypothesis was that there would be different expression of *MMP9* in DS and NDS patients. Subsequent analyses, dependent on the outcome of the primary hypothesis, explored the hypothesis that any such differences in DNA methylation of MMP9 would be associated with differences in gene expression and clinical symptoms.

## Materials and Methods

### Participants

A total of 104 patients with schizophrenia (51 DS and 53 NDS) and 50 healthy subjects participated in this study, based on approval by the Institutional Ethical Committee for clinical research of Wutaishan hospital, Jiangsu province, China. All participants were male Han Chinese, right-handed, and provided written informed consent. The patients with schizophrenia were recruited from the psychiatric rehabilitation unit of Yangzhou Wutaishan hospital. The inclusion criteria were (1) a diagnosis of schizophrenia according to Diagnostic and Statistical Manual of Mental Disorders, Fourth Edition (DSM-IV), and confirmed by the Chinese version of the Structured Clinical Interview for DSM-IV (SCID-I) (First et al., 1997); (2) age between 20 and 65 years; (3) long-standing psychiatric symptoms and stable antipsychotic pharmacological treatment for at least 12 months based on inpatient medical records. Exclusion criteria included any neurological or medical condition, such as head trauma, mental retardation, alcoholism or substance abuse, or a history of electroconvulsive therapy in the past 6 months. Deficit and non-deficit schizophrenia were diagnosed using the Chinese version of the Schedule for the Deficit Syndrome (SDS) (Wang et al., 2008). The healthy controls were selected from the community by matching patients with schizophrenia for age and handedness, excluding any Axis I psychiatric disorder of the Structured Clinical Interview for DSM-IV Non-Patient version (SCID-NP) (First et al., 1996) and with no family history of psychiatric disorders.

### Clinical Assessment of Patients

The Schedule for Deficit Syndrome (SDS) (Kirkpatrick et al., 1989) was used to categorize the patients with schizophrenia into DS and NDS groups, according to the assessment of six enduring (persistent over 12 months) and primary (instead of secondary sources) negative symptoms (restricted affect, diminished emotional range, poverty of speech, curbing of interests, diminished sense of purpose, diminished social drive). The Brief Psychiatric Rating Scale (BPRS), organized into separate positive, negative, disorganized and affect syndromes based on the findings of the factor analysis of 18-item (Mueser et al., 1997; Cohen et al., 2007), was used to evaluated a full range of symptomatology of DS and NDS patients. As BPRS fails to reflect the comprehensive range of negative symptoms, we utilized the Scale for the Assessment of Negative Symptoms (SANS, 19 original items) to assess the negative symptoms. The SANS scale was divided into three factors involving Diminished Expression (including items from the Affective Flattening or Blunting scale, as well as the “poverty of speech”), Inattention-Alogia (which included items from the Inattention and Alogia scales, as well as the “poor eye contact” item) and Social Amotivation (reflecting items from the Anhedonia-Asociality and Avolition-Apathy subscales) (Blanchard and Cohen, 2006; Lyne et al., 2013). Since attentional problems yield a poor fit related to the negative symptom construct (Peralta and Cuesta, 1995), the factor of Inattention-Alogia has not been included in exploratory analysis in the current study. The Scale for the Assessment of Positive Symptoms (SAPS) was used to assess positive symptoms.

### *MMP9* Expression and DNA Methylation Pyrosequencing Processing

Fasting venous blood samples were taken from the patients with schizophrenia and health subjects at 6–8 am in the morning. Peripheral blood mononuclear cells (PBMCs) were isolated from blood samples by using the BD Vacutainer Cell Preparation tubes according to the manufacturer’s instructions (Becton, Dickinson and Company, United States) and stored in the refrigerator by -80°C. Total RNA was isolated from PBMCs samples using RNeasy mini kit (Qiagen, CA, United States). The gene expression was obtained by using real-time quantitative PCR (RT-qPCR). cDNA sequences for human *MMP9* (forward: 5′-GTGGACGATGCCTGCAACGT-3′; reverse: 5′-GCCGCTCCTCAAAGACCGAG-3′) and *GAPDH* (forward: 5′-ACCACAGTCCATGCCATCAC-3′; reverse: 5′-TCCACCACCCTGTTGCTGTA-3′) were used for primer construction. cDNA samples were used for RT-qPCR experiment in duplicate. Real-time PCR was performed according to the manufacturer’s protocol using QuantiTect SYBR Green RT-PCR kit (Qiagen, United States). Briefly, 20 μL total reaction volume containing 10 μL SYBR Green master mix (Applied Biosystems), 0.1 μL each forward and reverse primer (10 pM/μL), and 2 μL cDNA was used in PCR using ABI 7900HT FAST instrument. PCR was performed with an initial incubation at 50°C for 2 min, followed by 10-min denaturation at 95°C and 40 cycles at 95°C for 15 s, 60°C for 1 min, and 72°C for 40 s. *MMP9* expression was normalized to the mRNA levels of housekeeping gene *GAPDH* (Barber et al., 2005). Delta–Delta CT (CT = threshold cycle) and relative mRNA levels of *MMP9* were calculated (Livak and Schmittgen, 2001). The relative fold changes of *MMP9* mRNA of DS or NDS group patients were compared with the mean *MMP9* mRNA of healthy subjects.

Genomic DNA was isolated from blood sample PBMCs using QIAamp DNA Blood Mini Kit (Qiagen, United States) and bisulfite-modified to convert unmethylated cytosine residues to uracil using EpiTec Fast DNA Bisulfite Kit (Qiagen, United States). PCR reactions were set up according to the instruction of PyroMark PCR Master Mix kit (Qiagen, Cat. No. 978703). In brief, gently mix 12.5 μl PyroMark PCR Master Mix, 2.5 μl CoralLoad Concentrate, 2 μl Primer, 6 μl RNase-free water and 2 μl template DNA. The thermal cycler is 95°C, 15 min; 94°C, 30 s, 56°C, 30 s, 72°C, 30 s, 45 cycles; 72°C, 10 min. After amplification, samples stored -20°C. Pyrosequencing was performed using the PyroMark Q96 ID System (Qiagen, United States) to analysis DNA methylation of *MMP9* gene in patients and healthy controls. According to CpG islands track of UCSC genome Browser^1^, we got the information that the human *MMP9* gene contains four CpG islands. In view of that DNA methylation usually occurs within promoter or nearby exon regions intragenically, we chose the sequence on the first CpG island containing exons 4 and 5 for analysis. The region containing exon 4 using Hs_MMP9_02_PM PyroMark CpG assay (Cat. No. PM00079198) analyzing sequence of 5′-GCCC**CG**GCATTCAGGGAGA**CG**CCCATTT**CG**A**CG**ATGA**CG**A-3′ and the region containing exon 5 using Hs_MMP9_01_PM PyroMark CpG assay (Cat. No. PM00079191) analyzing sequence of 5′-TCGGTTTGGAAACGCAGATGGCGCG-3′. Mean values of methylation of each exonic CpG-containing sequence were calculated. Totally, 9 CpG sites were included, naming CpG4-1, CpG4-2, CpG4-3, CpG4-4, CpG4-5, CpG5-1, CpG5-2, CpG5-3, and CpG5-4. The relative methylation changes of *MMP9* of DS or NDS group patients were compared with the mean *MMP9* methylation of healthy subjects (Gao et al., 2019).

### Statistical Analysis

Statistical analysis was undertaken using SPSS version 19.0 (SPSS Inc., United States). Data of demographic, clinical characteristics, gene expression and percentage methylation are presented as mean ± standard deviation. Differences between these three groups were determined through the use of ANCOVA analysis for continuous variables using education years as covariant, then Bonferroni *post hoc* analysis was used to compare between groups. Dichotomous variables (i.e., percentage smokers) were compared by chi-squared among three groups. Psychiatric symptoms between DS and NDS groups were compared using student’s t-tests. Spearman rank correlation and multiple linear regression were used to determine the influence of demographic factors, clinical measurements and gene methylation level on negative symptoms. Furthermore, partial correlation analyses were used to determine the relationships between CpG site methylation percentage of *MMP9* and clinical assessments controlling age and CPZ-equivalent variables. In testing our primary hypothesis, for the multiple comparisons between clinical groups for each CpG site, a Bonferroni-corrected *p*-value of 0.0056 was applied. Otherwise a two tailed *P*-value < 0.05 was predetermined as significant.

## Results

### Demographic and Clinical Characteristics

There was a significant difference in education years but not age nor the proportion of smokers among DS, NDS and HC groups. The DS group revealed fewer education years compared to HC subjects, while no significant difference was detected between the two schizophrenia subgroups. There was no significant difference in age at onset, duration and CPZ-equivalent dose between DS and NDS groups. DS patients showed significantly higher scores in negative syndrome than NDS, but not in positive syndrome, disorganization syndrome and affect factors of BPRS. Consistent with BPRS measurement, SANS scores were higher in DS group than NDS group but no significant differences seen in SAPS scores. DS patients showed significantly higher scores in the diminished expression and social amotivation factors of SANS than NDS, indicating that DS patients suffered more severe impairment in blunted affect, anhedonia and avolition (Table 1).

**Table 1:** Demographics and characteristics for deficit schizophrenia (DS), non-deficit schizophrenia (NDS), and healthy subjects (HC) groups.

	DS (*n* = 51)	NDS (*n* = 53)	HC (*n* = 50)	*F/χ^2^/t/Z*	*P*
Age	50.25 ± 6.91	48.15 ± 7.40	48.80 ± 6.55	1.235	0.294
Education years	7.53 ± 3.59^Δ^	8.51 ± 1.93	9.50 ± 2.57	6.390	0.002
Age at onset	21.69 ± 2.69	22.11 ± 2.63	NA	-0.818	0.415
Duration	28.57 ± 6.69	26.04 ± 7.57	NA	1.804	0.074
Percentage smokers (%)	61	77	78	4.838	0.089
BPRS total score	32.63 ± 2.85^∗∗^	27.55 ± 3.03	NA	8.792	<0.001
Positive syndrome	6.22 ± 1.05	6.23 ± 1.09	NA	-0.051	0.959
Negative syndrome	12.98 ± 1.88^∗∗^	7.53 ± 1.31	NA	17.090	<0.001
Disorganized syndrome	6.47 ± 1.24	6.51 ± 0.95	NA	-0.442	0.660
Affect	6.96 ± 1.06	7.23 ± 1.35	NA	-1.117	0.267
SAPS total score	9.10 ± 3.74	9.32 ± 4.37	NA	-0.279	0.781
SANS total score	57.71 ± 8.14^∗∗^	32.72 ± 7.20	NA	15.228	<0.001
Diminished expression	17.33 ± 3.56^∗∗^	9.15 ± 2.08	NA	14.382	<0.001
Social amotivation	15.35 ± 2.66^∗∗^	9.21 ± 2.64	NA	11.820	<0.001
Expression + Amotivation	32.69 ± 4.94^∗∗^	18.36 ± 4.18	NA	15.929	<0.001
CPZ-equivalent dose; mg/day (IQR)	450 (300–600)	500 (400–610)	NA	-1.263	0.207

*Values represented as mean ± S.D.*

*DS, deficit schizophrenia; NDS, non-deficit schizophrenia; HC, healthy controls; BPRS, Breif Psychiatric Rating Scale; SAPS, the Scale for the Assessment of Positive Symptoms; SANS, the Scale for the Assessment of Negative Symptoms; CPZ, chlorpromazine; IQR, interquartile range.*

*^∗∗^P < 0.001 DS vs. NDS.*

*(^Δ^P ( <0.01 DS vs. HC.*

### Methylation of the *MMP9* and Gene Expression in PBMCs

Mean methylation levels of nine sites in the *MMP9* were significantly different among DS, NDS and HC groups. Compared to NDS patients, DS had significantly lower methylation levels in CpG4-4, CpG4-5, CpG5-1, CpG5-2, CpG5-3, CpG5-4 (all adjusted *P* < 0.001), while CpG4-2 (*P* = 0.027), CpG4-3 (*P* = 0.262) showed no significant difference and CpG4-1 (adjusted *P* < 0.001) had a higher level. *Post hoc* analysis showed DNA methylation of all individual sites in both DS and NDS patients were significant lower than HC subjects, except that DNA methylation of CpG5-3 did not differ between NDS and HC (*P* = 0.027). Mean values of DNA methylation were significantly different in exons 4 and 5 of *MMP9* among three groups. Bonferroni *post hoc* comparisons revealed lower methylation levels in both exons 4 and 5 in DS patients (*P* < 0.001) and NDS (*P* < 0.001) relative to HC subjects, while DS patients showed significantly lower DNA methylation of exons 4 and 5 than NDS. Repeating the initial analysis with smoking status and CPZ equivalents included as an additional factor and covariate, respectively, showed essentially equivalent results with no differences in the levels of significance reached for each CpG site (data not shown).

The gene expression of *MMP9* in peripheral blood mononuclear cells showed significant higher levels in DS and NDS patients than HC subjects and it was higher in DS patients than NDS patients (Table 2). Here too neither smoking status nor CPZ equivalents influenced these findings.

**Table 2:** Methylation of MMP-9 and gene expression in DS, NDS, and HC group.

	DS	NDS	HC	*F*	*P*	*Cohen’ d*
	(*n* = 51)	(*n* = 53)	(*n* = 50)			_(DS vs. NDS)_
*MMP9*^Δ^	1.73 ± 0.68*##	1.37 ± 0.43#	1.06 ± 0.37	21.576	<0.001	0.633
CpG4-1 (%)	7.86 ± 0.94**##	5.60 ± 0.79##	9.92 ± 0.92	306.228	<0.001	2.603
CpG4-2 (%)	5.12 ± 0.79##	4.77 ± 0.72##	8.36 ± 0.75	333.088	<0.001	NS
CpG4-3 (%)	7.55 ± 0.78##	7.75 ± 0.96##	9.76 ± 1.00	89.305	<0.001	NS
CpG4-4 (%)	5.67 ± 0.93**##	7.64 ± 0.74##	9.18 ± 0.92	209.692	<0.001	2.344
CpG4-5 (%)	3.35 ± 0.72**##	6.60 ± 0.86##	8.04 ± 0.97	400.453	<0.001	4.098
CpG5-1 (%)	2.84 ± 0.58**##	4.77 ± 0.80##	5.66 ± 0.85	186.287	<0.001	2.762
CpG5-2 (%)	3.45 ± 0.61**##	4.23 ± 0.70##	5.32 ± 0.96	75.658	<0.001	1.188
CpG5-3 (%)	3.76 ± 0.76**##	5.17 ± 0.73	4.84 ± 0.79	48.108	<0.001	1.892
CpG5-4 (%)	4.10 ± 0.81**##	6.08 ± 0.76##	7.20 ± 1.26	134.471	<0.001	2.521
Exon 4 (%)	5.91 ± 0.48**##	6.48 ± 0.39##	9.05 ± 0.62	553.223	<0.001	1.303
Exon 5 (%)	3.54 ± 0.49**##	5.06 ± 0.52#	5.76 ± 0.65	211.032	<0.001	3.008

*^∗^P < 0.01 DS vs. NDS; ^∗∗^P < 0.001 DS vs. NDS; ^#^P < 0.01 DS/NDS vs. HC; ^##^P < 0.001 DS/NDS vs. HC.*

*Values are expressed as % methylation.*

*^Δ^Relative fold changes of MMP9 (range: DS, 0.87–2.85; NDS, 0.74–2.23; HC, 0.36–1.70).*

### Relationship Between *MMP9* Methylation, Gene Expression, Demographic and Clinical Characteristics

**FIGURE 1:**
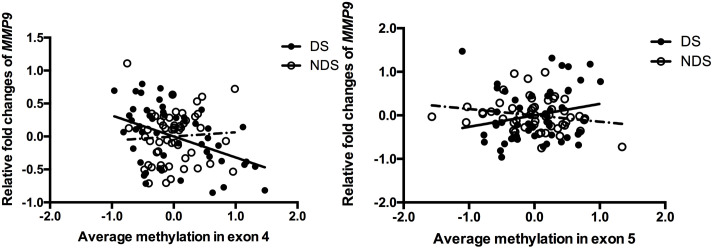
Correlation between *MMP9* expression and methylation in exons 4 and 5. The points of the scatter plot represented residual value of mean methylation of exon 4/exon 5 and relative fold changes of *MMP9* (gene expression) after controlled covariates in DS (filled dots) and NDS (empty dots) groups. The mean methylation was calculated by using the relative methylation changes in each CpG site.

Stepwise linear regression indicated a significant effect of age (β = 0.031, *P* = 0.02) and medicine (CPZ-equivalent dose) (β = 0.001, *P* = 0.029) on gene expression of MMP9 in DS group, but no significant effect of education, duration, onset and smoking status. After controlling for age and CPZ-equivalent dose, DNA methylation in exon 4 (*r* = -0.388, *P* = 0.005) but not exon 5 (*r* = 0.252, *P* = 0.074) remained significantly negatively correlated with gene expression of MMP9 in DS group (Figure 1). Age had a significant effect (β = 0.026, *P* < 0.001) on gene expression of MMP9 in NDS group, while other variables showed no significant effect. Controlling for age effect, there was no significant correlation between gene expression and methylation in both exon 4 (*r* = 0.163, *P* = 0.244) and exon 5 (*r* = -0.225, *P* = 0.106) in NDS group (Figure 1). There was no significant correlation between gene expression of MMP9 and average methylation in exon 4 or exon 5 in HC group.

### Relationship Between Clinical Symptoms and *MMP9* Methylation

**FIGURE 2:**
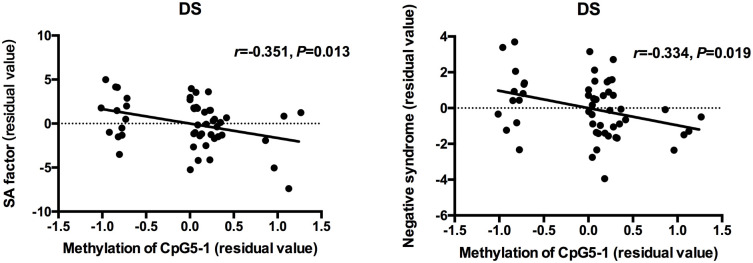
Correlation between DNA methylation of MMP9 and assessment of clinical symptoms. The points of the scatter plot represented residual value of DNA methylation of CpG5-1 and subscale of SANS and BPRS after controlled age and CPZ-equivalent as covariates in DS patients.

Partial correlation analysis was used to determine the relationships between clinical measurements, *MMP9* DNA methylation and expression in patients when controlling for age and CPZ-equivalent. For all patients with schizophrenia, a significant positive correlation was found between gene expression of MMP9 and negative symptoms (SANS total scores: *r* = 0.256 *P* = 0.009 and negative syndrome subscale of BPRS: *r* = 0.272 *P* = 0.006), but no significant correlation was found with positive symptoms. The lower methylation level of individual CpG sites was associated with higher SANS scores except for CpG4-1, CpG4-2, and CpG4-3. After dividing patients into subgroups, the social amotivation factor of SANS (*r* = -0.351, *P* = 0.013) and negative syndrome of BPRS (*r* = -0.334, *P* = 0.019) was negative correlated with DNA methylation of CpG5-1 in DS patients but not in NDS patients (Figure 2). The correlation diagrams were presented with the residual values of CpG methylation and clinical assessments.

## Discussion

The present study demonstrated that the epigenetic pattern and gene expression of MMP9 of peripheral blood mononuclear cells was different among DS, NDS, and HC groups. DS patients had an increased gene expression of MMP9, which might be relative with the lower level of average methylation in exon 4, near the promoter region of the *MMP9*. Notably, DNA methylation of individual CpG sites showed negative correlations with clinical symptoms such as the social amotivation factor of SANS and negative syndromes of BPRS in DS patients, but not in NDS patients. To the best of our knowledge, this was the first study to provide evidence of DNA methylation involved in gene expression of MMP9 in patients with schizophrenia, contributing to our understanding of the potential pathogenesis of DS patients.

MMP9 has been considered to have pathological importance in patients with schizophrenia (Lepeta and Kaczmarek, 2015). Domenici et al. (2010) applied a focused proteomic approach in a large scale case-control study including 229 schizophrenic patients and 254 controls and revealed increased peripheral MMP9 in patients with schizophrenia. Similar results were reported by the recent ROC curve analysis (Ali et al., 2017) indicating that the increased MMP9 had some value in distinguishing schizophrenia and healthy controls. Increased peripheral MMP9 was also reported in remitted (Devanarayanan et al., 2015) and treatment-resistant schizophrenia (Yamamori et al., 2013). However, a few studies have shown negative findings of peripheral MMP9 in patients with schizophrenia, which had been attributed to disease state, medicine treatment (Kumarasinghe et al., 2013) or smoking status (Niitsu et al., 2014). For example, Kumarasinghe et al. (2013) found that *MMP9* mRNA was significantly up-regulated in PBMCs of treatment-naïve schizophrenic patients than healthy subjects and returned to control level after 6–8 weeks antipsychotic pharmacotherapy of 200 mg/d CPZ-equivalents. Our study was consistent with the majority of the previous reports (Domenici et al., 2010; Devanarayanan et al., 2015; Ali et al., 2017) showing an increase *MMP9* expression in PBMCs in these long-term stabilized patients with schizophrenia. This study along with our recent study (Gao et al., 2019) also indicated that MMP9 was significantly elevated in DS patients relative to NDS patients. As influencing factors including age, gender and smoking status were well matched, the increased MMP9 observed in the present study might reflect an association with clinical symptoms, especially the primary and persistent negative symptoms in DS patients compared with NDS patients.

A positive correlation was found between gene expression of MMP9 and negative symptoms after controlling for age and CPZ-equivalents in the total schizophrenia group, suggesting the increased MMP9 might have a potential effect on negative symptoms. MMP9 is synthesized by neurons, astrocytes and microglia in hippocampal and prefrontal cortex (Okulski et al., 2007), which are the critical brain regions associated with negative symptoms in schizophrenia (Shaffer et al., 2015; Palm et al., 2016). Altered LTP in *MMP9* knockout mice was first reported by Nagy et al. (2006), indicating that MMP9 plays an important role in hippocampal synaptic physiology and plasticity. This finding has been supported by several other studies, including investigation of different pathways of LTP in hippocampus. Wiera et al. (2013) reported that the maintenance of LTP was nearly abolished in the mossy fiber-CA3 projection of hippocampus in both the MMP9 overexpressing and the *MMP9* knockout rats. Abnormal synaptic plasticity renders the patient incapable of learning from social experiences and adaptive exchanges, which is likely to trigger social withdrawal and apathy in schizophrenia (Stephan et al., 2009). Moreover, Michaluk et al. (2009) reported that MMP9 stimulated the surface trafficking of NMDAR through increasing the lateral diffusion in hippocampal neurons of rats *in vitro*. The lateral diffusion was found as the key process of NMDA receptor internalization (Groc et al., 2009), indicating that the increased MMP9 might theoretically lead to NMDAR hypoactivity in hippocampus. Studies focusing on the relationship between MMP9 and NMDAR in the prefrontal cortex are virtually absent. It is well known that NMDAR hypoactivity in the prefrontal cortex contributes to the inhibition of the dopamine pathway from ventral tegmental area to dorsolateral and ventromedial prefrontal cortex (Stahl, 2007). Therefore, it might be speculated that the elevated MMP9 might be involved in functional disturbances of NMDA receptor in the hippocampus and prefrontal cortex and possibly be associated with negative symptoms and cognitive dysfunction in schizophrenia. Additionally, it should be noted that the present study found no significant correlation between SANS scores and gene expression of *MMP9* in either the DS or NDS group. Whether this negative finding could be attributed to ceiling effects of severe negative symptoms in two patient groups remains unclear.

The association between gene polymorphism and behavioral symptoms in schizophrenia might provide a mechanistic insight into *MMP9* gene function. Recently, a phenotype-based genetic association study by Lepeta et al. (2017) demonstrated an association between the *MMP9* rs20544 CC/CT genotype and severity of a chronic delusional syndrome. The molecular mechanism might be the rs20544 C/T single-nucleotide polymorphism (SNP) affects the affinity of Fragile X mental retardation protein (FMRP) binding to MMP9 mRNA and influences MMP9 activity at dendritic spines. According to exposing the *MMP9* heterozygous mice to psychosis-related locomotor hyperactivity induced by an NMDA receptor antagonist, they also indicated the lower MMP9 level influenced performance in a behavioral model of the positive symptoms of schizophrenia (Lepeta et al., 2017). Another research reported by Bienkowski et al. (2015) has found no association between the functional *MMP9* -1562C/T gene polymorphism and deficit/non-deficit subtypes of schizophrenia, which seemed to argue against the role of *MMP9* gene polymorphism in deficit symptomatology. However, one should be aware that presumed genetic differences between deficit and non-deficit subtypes may involve many genes and that the influence of a single gene can be relatively weak and difficult to confirm. The present study employed different methodologies (e.g., *MMP9* expression and methylation) to study deficit and non-deficit subtypes and further demonstrated the association between elevated gene expression of MMP9 and negative symptoms of schizophrenia. Thus gene polymorphism and alterations of MMP9, both down- as well as up-regulated, might be possible factors contributing to the pathophysiological underpinning of schizophrenia.

The present study demonstrated hypo-methylation status in exon 4 and exon 5 of *MMP9* in two schizophrenia subgroups compared to health controls, which might result in up-regulated gene expression of MMP9. Previous studies demonstrated that gene expression of MMP9 might be mediated by DNA methylation in *MMP9* promoter and intragenic DNA regions, indicating a mechanism of regulation by epigenetic modifications (Campos et al., 2016; Klassen et al., 2018). Roach et al. (2005) showed that demethylation of the 5′-flanking region containing the promoter of *MMP9* was associated with elevated expression patterns of MMP9 in human osteoarthritic chondrocytes (Roach et al., 2005). Zybura-Broda et al. (2016) identified gene expression was regulated by demethylation of *MMP9* proximal promoter in human epilepsy. Aforementioned studies indicated an important role of epigenetic mechanism played in *MMP9* up-regulation and protease activity, also that might be implicated in the MMP9 biological process in schizophrenia. DNA methylation, one of the principal epigenetic mechanisms, has been importantly implicated in the pathophysiology of schizophrenia (Feng and Fan, 2009; Kinoshita et al., 2013). The present study provided the first evidence showing the possible association between DNA methylation and gene expression of MMP9 in schizophrenia. Importantly, the DS patients showed lower methylation than NDS patients at the majority of individual CpG sites, while a negative correlation was found between gene expression and average methylation of exon 4 for *MMP9* in DS patients. DS patients had poorer premorbid adjustment during childhood and early adolescence and exhibited more impairment in general cognitive abilities than NDS patients (Galderisi et al., 2002). Considering that MMP9 apparently plays a special role during developmental plasticity, it would raise an intriguing assumption that the hypo-methylation pattern of MMP9 might affect downstream cellular change and contribute to the manifestation of behavioral abnormalities or clinical symptoms from the early life of DS patients.

Lower methylation at several individual CpG sites and higher *MMP9* expression was associated with higher SANS scores in schizophrenia patients in the present study. This interesting finding is in keeping with the expectation that lower methylation would be associated with increased MMP9, which in turn could contribute to the neuropathology that may underlie negative symptomatology. Generally, promoter sequence methylation could directly interfere with transcription factor binding sites or indirectly cause gene silencing through methylation DNA binding proteins that recruit histone deacetylases, leading to chromatin condensation (Jones et al., 1998). Predicted by bioinformatics methods (PROMO v8.3) (Messeguer et al., 2002), the region of *MMP9* adjacent to CpG4-4, CpG4-5 and CpG5-1 contains recognition sequences of several important transcription factors, such as p53, GR-alpha and c-Myb. p53 is importantly involved in proliferation, differentiation and apoptosis of neural progenitor cells (Tedeschi and Di Giovanni, 2009). Thus the hypo-methylation in exon 4 of *MMP9* might possibly enhance the activity of transcription factors (e.g., p53), and thereby increase gene expression of MMP9 in DS patients. Notably, the present study further found that both the social amotivation factor of SANS and the negative syndrome of BPRS were negatively correlated with the DNA methylation of CpG5-1 particularly in DS patients, indicating a distinct epigenetic characteristic of DS patients. The social amotivation factor of SANS would represent the behavioral abnormality relevant to the learning disability of social experiences and adaptive exchanges (Stephan et al., 2009), consistent with the hypothesis that MMP9 contributes to pathological synaptic plasticity in schizophrenia (Lepeta and Kaczmarek, 2015). The present findings thus provide a possible linkage between hypo-methylation of *MMP9* and negative symptoms in DS patients.

Several limitations of the present study should be considered. The main limitation was that MMP9 DNA methylation and gene expression were detected in PBMCs, where MMP9 expression and methylation may not necessarily represent that occurring in brain regions relevant to the pathogenesis of schizophrenia. However, several previous reports have also demonstrated that MMP9 expression is substantially altered in peripheral blood in patients with schizophrenia. The second limitation is that the present study did not completely analyze the DNA methylation at full length of promoter (exon) region of MMP9. The promoter (exon1) region, containing many CpG sites, was omitted in the present study, although this region does not overlap with a CpG island. Further study is needed to reveal a more comprehensive understanding of MMP9 DNA methylation in DS. Thirdly, the present study used CPZ-equivalent daily dose as the parameter to evaluate antipsychotic medication for the recruited patients, wherein the type and dosage of antipsychotic drug was comparable between the two patient groups. The influence of antipsychotic medication on MMP9 expression and methylation does not here differentiate typical and atypical antipsychotics, although differential epigenetic modification of various antipsychotics in the patients with schizophrenia has been reported (Ibi and Gonzalez-Maeso, 2015). Further studies would be needed to determine the distinct epigenetic effects on the MMP9 following different antipsychotic medication. Finally, the present study is limited in the sample sizes of the DS, NDS and control groups. This is an inevitable consequence of the strict diagnostic criteria for DS and the need to restrict variance by eliminating or minimizing confounders such as age, gender, smoking and fluctuations of psychiatric symptoms. Replication with a larger sample size would be valuable to increase the statistical power and fully investigate the effects of various clinical confounders.

In summary, the present study provided evidence for abnormal peripheral gene expression and DNA methylation of MMP9 in DS patients, indicating that subjects with the deficit syndrome might be a specific sub-group within schizophrenia. The negative correlations between *MMP9* DNA methylation of individual CpG sites and negative symptoms revealed a distinct neuropathological impairment in DS patients. The present study indicated that *MMP9* methylation might be a promising disease biomarker especially for the diagnosis and treatment domains of negative symptoms.

## Ethics Statement

This study was carried out in accordance with the recommendations of ‘name of guidelines, name of committee’ with written informed consent from all subjects. All subjects gave written informed consent in accordance with the Declaration of Helsinki. The protocol was approved by the ‘name of committee’.

## Author Contributions

JG and HY performed the research and analyzed the data. JG and XT wrote the manuscript. XgZ made substantial contributions to conception and coordination. XF, MY, WS, and XbZ help collecting samples. XW provided the scale for assess clinical symptoms. All authors read and approved the final manuscript.

## Conflict of Interest Statement

The authors declare that the research was conducted in the absence of any commercial or financial relationships that could be construed as a potential conflict of interest.

## Footnotes

1 http://genome.ucsc.edu/
