# Corrigendum: DNA Methylation and Gene Expression of Matrix Metalloproteinase 9 Gene in Deficit and Non-deficit Schizophrenia

**DOI:** 10.3389/fgene.2020.00823

**Published:** 2020-08-14

**Authors:** Ju Gao, Hongwei Yi, Xiaowei Tang, Xiaotang Feng, Miao Yu, Weiwei Sha, Xiang Wang, Xiaobin Zhang, Xiangrong Zhang

**Affiliations:** ^1^Department of Geriatric Psychiatry, Nanjing Brain Hospital, Affiliated to Nanjing Medical University, Nanjing, China; ^2^Centers of Disease Prevention and Control for Mental Disorders, Shanghai Changning Mental Health Center, Shanghai, China; ^3^Department of Pharmacology, School of Medicine, Southeast University, Nanjing, China; ^4^Department of Psychiatry, Affiliated WuTaiShan Hospital of Medical College, Yangzhou University, Yangzhou, China; ^5^Department of Psychiatry, Nanjing Qing Long Mountain Psychiatric Hospital, Nanjing, China; ^6^Medical Psychological Institute of the Second Xiangya Hospital, Central South University, Changsha, China

**Keywords:** deficit schizophrenia, matrix metalloproteinase-9, DNA methylation, gene expression, negative symptoms, pyrosequencing

This corrigendum is to declare that the preliminary analysis of the data on *MMP9* DNA methylation in the present article was published in the Letter to the Editor published in Schizophrenia Research. While the main focus, results and conclusions of the two papers differ, the data of *MMP9* DNA methylation in Table 2 of the present article and the data in Figure 1B of the letter to editor in Schizophrenia Research represent essentially the same data, apart from a small difference in the numbers of cases. In order to avoid misinterpretation, we would like to add the citation of Gao et al. (2019) in the following places:

The **Materials and Methods** section, subsection **MMP9 Expression and DNA Methylation Pyrosequencing Processing**, pargraph 2:

“Genomic DNA was isolated from blood sample PBMCs using QIAamp DNA Blood Mini Kit (Qiagen, United States) and bisulfite-modified to convert unmethylated cytosine residues to uracil using EpiTec Fast DNA Bisulfite Kit (Qiagen, United States). PCR reactions were set up according to the instruction of PyroMark PCR Master Mix kit (Qiagen, Cat. No. 978703). In brief, gently mix 12.5 μl PyroMark PCR Master Mix, 2.5 μl CoralLoad Concentrate, 2 μl Primer, 6 μl RNase-free water and 2 μl template DNA. The thermal cycler is 95°C, 15 min; 94°C, 30 s, 56°C, 30 s, 72°C, 30 s, 45 cycles; 72°C, 10 min. After amplification, samples stored −20°C. Pyrosequencing was performed using the PyroMark Q96 ID System (Qiagen, United States) to analysis DNA methylation of *MMP9* gene in patients and healthy controls. According to CpG islands track of UCSC genome Browser, we got the information that the human *MMP9* gene contains four CpG islands. In view of that DNA methylation usually occurs within promoter or nearby exon regions intragenically, we chose the sequence on the first CpG island containing exons 4 and 5 for analysis. The region containing exon 4 using Hs_MMP9_02_PM PyroMarkCpG assay (Cat. No. PM00079198) analyzing sequence of 5′-GCCCCGGCATTCAGGGAGACGCCCATTTCGACGATGACGA-3′ and the region containing exon 5 using Hs_MMP9_01_PM PyroMark CpG assay (Cat. No. PM00079191) analyzing sequence of 5′-TCGGTTTGGAAACGCAGATGGCGCG-3′. Mean values of methylation of each exonic CpG-containing sequence were calculated. Totally, 9 CpG sites were included, naming CpG4-1, CpG4-2, CpG4-3, CpG4-4, CpG4-5, CpG5-1, CpG5-2, CpG5-3, and CpG5-4. The relative methylation changes of *MMP9* of DS or NDS group patients were compared with the mean *MMP9* methylation of healthy subjects (Gao et al., 2019).”

The **Discussion** section, paragraph 2:

“MMP9 has been considered to have pathological importance in patients with schizophrenia (Lepeta and Kaczmarek, 2015). Domenici et al. (2010) applied a focused proteomic approach in a large scale case-control study including 229 schizophrenic patients and 254 controls and revealed increased peripheral MMP9 in patients with schizophrenia. Similar results were reported by the recent ROC curve analysis (Ali et al., 2017) indicating that the increased MMP9 had some value in distinguishing schizophrenia and healthy controls. Increased peripheral MMP9 was also reported in remitted (Devanarayanan et al., 2015) and treatment-resistant schizophrenia (Yamamori et al., 2013). However, a few studies have shown negative findings of peripheral MMP9 in patients with schizophrenia, which had been attributed to disease state, medicine treatment (Kumarasinghe et al., 2013) or smoking status (Niitsu et al., 2014). For example, Kumarasinghe et al. (2013) found that *MMP9* mRNA was significantly up-regulated in PBMCs of treatment-naïve schizophrenic patients than healthy subjects and returned to control level after 6–8 weeks antipsychotic pharmacotherapy of 200 mg/d CPZ-equivalents. Our study was consistent with the majority of the previous reports (Domenici et al., 2010; Devanarayanan et al., 2015; Ali et al., 2017) showing an increase *MMP9* expression in PBMCs in these long-term stabilized patients with schizophrenia. This study along with our recent study (Gao et al., 2019) also indicated that MMP9 was significantly elevated in DS patients relative to NDS patients. As influencing factors including age, gender and smoking status were well matched, the increased MMP9 observed in the present study might reflect an association with clinical symptoms, especially the primary and persistent negative symptoms in DS patients compared with NDS patients.”

The authors apologize for not being able to accurately reference the Letter to the Editor due to the close publication date of the two articles and state that this does not change the scientific conclusions of the article in any way. The original article has been updated.
